# Community Detection Method Based on Node Density, Degree Centrality, and K-Means Clustering in Complex Network

**DOI:** 10.3390/e21121145

**Published:** 2019-11-23

**Authors:** Biao Cai, Lina Zeng, Yanpeng Wang, Hongjun Li, Yanmei Hu

**Affiliations:** 1 College of Information Science &Technology, Chengdu University of Technology, Chengdu 610059, China; 2 Key Laboratory of Manufacturing Process Testing Technology of Ministry of Education of China, Southwest of University of Science and Technology, Mianyang 621010, China

**Keywords:** community detection, CB-uncertainty (Community belongings uncertainty), DD (the combination of node density and node degree centrality), *k*-means

## Abstract

Community detection in networks plays a key role in understanding their structures, and the application of clustering algorithms in community detection tasks in complex networks has attracted intensive attention in recent years. In this paper, based on the definition of uncertainty of node community belongings, the node density is proposed first. After that, the DD (the combination of node density and node degree centrality) is proposed for initial node selection in community detection. Finally, based on the DD and *k*-means clustering algorithm, we proposed a community detection approach, the density-degree centrality-jaccard-*k*-means method (DDJKM). The DDJKM algorithm can avoid the problem of random selection of initial cluster centers in conventional *k*-means clustering algorithms, so that isolated nodes will not be selected as initial cluster centers. Additionally, DDJKM can reduce the iteration times in the clustering process and the over-short distances between the initial cluster centers can be avoided by calculating the node similarity. The proposed method is compared with state-of-the-art algorithms on synthetic networks and real-world networks. The experimental results show the effectiveness of the proposed method in accurately describing the community. The results also show that the DDJKM is practical a approach for the detection of communities with large network datasets.

## 1. Introduction

Recently, complex networks have attracted a great deal of attention in various fields [1,2], including sociology, computer science, mathematics, and biology. For large-scale networks, the presence of communities is an important feature, as it indicates the existence of groups of vertices within which connections are dense, but between which they are sparse [3]. Indeed, community detection has been widely applied in, e.g., community establishment in social media [4], the collection of similar features in parallel processing [5,6], and sharing research interests by intergroup authors in co-authorship networks [7].

To date, a large number of community detection algorithms for complex networks have been proposed [8,9], including hierarchical clustering algorithms [10], label propagation algorithms [11,12,13], density-based algorithms [14,15], random-walk-based algorithms [16,17], and so on. The *k*-means clustering algorithm divides the data into clusters (the cluster number is predetermined) based on minimum error functions [18]. This algorithm is characterized by rapid clustering, easy implementation, and effective classification in large-scale dataset, and has been widely applied for community detection in complex networks. Additionally, the *k*-means clustering algorithm shows low time complexity compared to clustering methods based on centrality and similarity [19,20,21]. Nevertheless, conventional *k*-means clustering algorithms have several limitations [22]. First, the selection of initial cluster centers in traditional *k*-means clustering algorithms, which has a determining effect on the clustering result, is a random process. Hence, effective clustering cannot be guaranteed [23]. Second, the node similarity has a significant effect on the convergence rate and accuracy of *k*-means clustering algorithms. Therefore, the iteration times in the *k*-means clustering algorithm can be effectively reduced, and the accuracy of community classification can be effectively improved by selecting appropriate initial cluster centers, defining appropriate node similarities, and setting appropriate stop conditions.

In this paper, the *k*-means clustering-based DDJKM algorithm for community detection was proposed, in which the community belongingness of nodes was described by the node uncertainty; density was introduced by information entropy, and the initial cluster centers were selected by the balance of the degree centrality, density, and the similarity of nodes. In this algorithm, the node similarity matrix is constructed as the clustering matrix by the node similarity in the network. This algorithm can effectively select the clustering center, thus preventing the selection of initial cluster centers that are too close to each other, and reducing the iteration times in the clustering process. The experimental results show the feasibility of the algorithm.

The rest of the paper is organized as follows: The theory behind the proposed algorithm, including the calculation equations for node uncertainty, node degree, node density, node balance, and node similarity, is discussed in Section 2. The details of DDJKM algorithm are given in Section 3. The performance of the proposed algorithm is evaluated in real-world networks and artificial networks, and compared with those of existing algorithms in Section 4. Finally, the conclusion is presented in Section 5.

## 2. Theory

### 2.1. Uncertainty

In the study of community structures in complex networks, the community belongingness (CB) of a node is certain if this node and its adjacent nodes are in the same community. Otherwise, the CB of a given node exhibits uncertainty. This is consistent with evaluations of information uncertainty by information entropy, where information uncertainty is proportional to information entropy. Therefore, the uncertainty of CB of nodes was established as follows:

The network is represented by an unweighted, undirected graph G= (V, E) , where $V\left(G\right)=\left\{v_{1},\;v_{2},\;\ldots ,\;v_{n}\right\}$ refers to the node set, and $E\left(G\right)=\left\{e_{1},\;e_{2},\;\ldots ,\;e_{k}\right\}$ refers to the edge set. |V|=n, |E|=m . $N\left(v_{i}\right)$ refers to the neighbor node set in the subgraph generated by the h hops forward breadth-first search (BFS) of $v_{i}\;$. If all $N\left(v_{i}\right)$ are in community $c_{j}$, the CB uncertainty of $v_{i}$ in $c_{j}$ is 0; if the majority of $N\left(v_{i}\right)$ are in community $c_{j}\;$, the CB uncertainty of $v_{i}$ in $c_{j}$. is considered to be low; if the majority of $N\left(v_{i}\right)$. are not in community *c_j_*, the CB uncertainty of $v_{i}$ in $c_{j}$ is considered to be high. The parameter *m* refers to the number of communities in the network, and the CB uncertainty of the node refers to a quantified parameter if the node does not belong to a specific community. The CB uncertainty of a node in a specific community is defined as a random variable $C\left(c_{1},c_{2},c_{3},\ldots ,c_{m}\right)$, and the probability of the i -th node in the q -th community is defined as $p\left(c_{q}\right)\;$, where q=1,2,…,m . Then, the CB uncertainty of $v_{i}$ is defined as:

(1) $$Entropy\left(v_{i}^{h}\right)=-\sum_{q=1}^{m}p\left(c_{q}\right)\log_{2}p\left(c_{q}\right)$$

where i refers to the node number, h refers to the forward hops of BFS, and $G_{i}^{h}$ refers to the subgraph generated by h-hop BFS of $v_{i}$ as the initial node respectively. $p\left(c_{q}\right)$ refers to the ratio of the number of nodes in the subgraph $G_{i}^{h}\left(N\right)$ to the number of nodes in the community $c_{q}\left(N^{\prime }\right)\;$:

(2) $$p\left(c_{q}\right)=\frac{N^{\prime }}{N}$$

**Figure 1:**
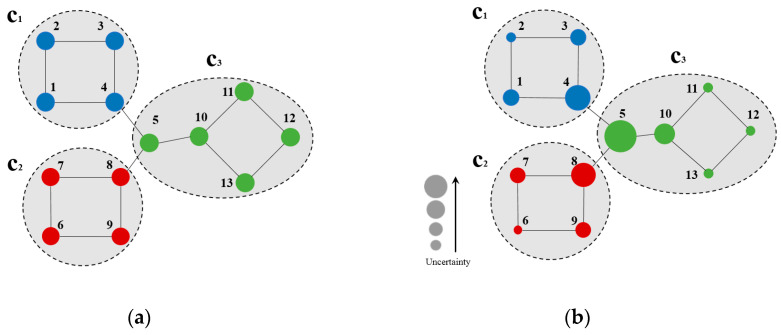
(**a**) Sample network; (**b**) Node uncertainty on the sample network at *h* = 2.

Figure 1 describes the CB uncertainty of example nodes. As shown in Figure 1a, three communities $\left(c_{1},\;c_{2},\;c_{3}\right)$ were presented, node 2 was identified in $c_{1}\;$, as well as nodes 1–4 in its subgraph of node 2 generated by two-hop forward BFS. According to Equation (2), the quantity ratios of nodes in the subgraph generated by a two-hop forward BFS of node 2 that are in *c*_1_, *c*_2_, and *c*_3_ and all nodes in the subgraph were $p(c_{1})=1,\;p(c_{2})=0,\mathrm{and}\;p(c_{3})=0\;$, respectively. The uncertainty of node 2 at *h* = 2 was calculated by Equation (1):

$$Entropy\left(v_{2}^{2}\right)=-\left(1\times \log_{2}1+0+0\right)=0$$

Figure 1b shows the uncertainty of nodes on the sample network (node uncertainty decreased with its size). According to Figure 1b, nodes with high uncertainty are marginal ones connected to the community (e.g., nodes 4, 5, 8, and 10 in Figure 1b). Herein, node 5 exhibits maximum uncertainty, as it is connected to all three communities. On the other hand, nodes with low uncertainty are marginal ones that are not adjacent to any other community (e.g., nodes 2, 6, and 11–13 in Figure 1b), as the community belongingness of these nodes is highly likely.

### 2.2. Community Belongingness

To determine the CB uncertainty of a given node, it is essential to obtain the CB of the node in advance. However, the initial CB of nodes for community detections in complex networks is unknown, and the CB uncertainty of nodes cannot be used as criteria for the selection of initial nodes in community detection algorithms; instead, quantified evaluation of the CB certainty of the corresponding node is required. As density is a measurable parameter in nature, we propose that the selection of initial nodes for community detection shall be based on the node density, instead of the entropy in the network. The node density is determined based on quantities of edges and nodes in the subgraph generated by a h-hop forward BFS of this node; it quantifies the CB certainty of this node in a specific community. The node density is defined as:

(3) $$Density\;\left(v_{i}^{h}\right)=\left|E^{\prime }\right|/\left(\left|V^{\prime }\right|\left(\left|V^{\prime }\right|-1\right)/2\right)$$

where i refers to the i -th node, h refers to the forward hop count from $v_{i}\;$, $V^{\prime }$ refers to the set of nodes in the subgraph $G^{\prime }$ with h hops forward BFS from $v_{i}\;$, |$V^{\prime }\;$| refers to the quantity of nodes in $V^{\prime }\;$, E′ refers to the set of edges in the subgraph $G^{\prime }\;$, and |E′ | refers to the quantity of edges in E′ .

**Figure 2:**
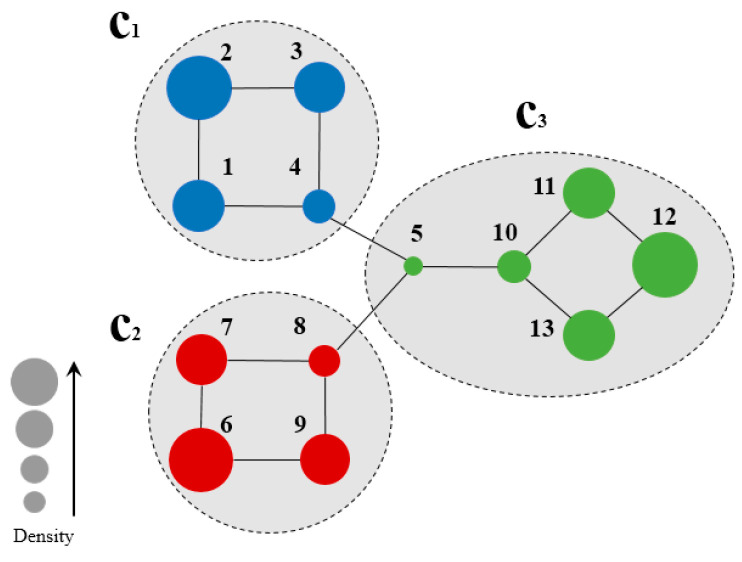
Community Belongingness of each node on the sample network of Figure 1a when *h* = 2.

Figure 2 shows a sample network for the calculation of node density, and Table 1 summarizes the node density of the two-hop subgraph of each node.

Figure 2 illustrates a sample network for the calculation of node density. Herein, a two-hop forward was involved due to the small size of the sample network. For example, from the calculation of the density of node 1, the set of nodes two hops forward from node 1 is:

$$n(v_{i}^{h})=n(v_{1}^{2})=\left\{1,\;2,\;3,\;4,\;5\right\}.$$

Five nodes and five edges were observed in the subgraphs. The density of node 1 can be calculated by Equation (3):

$$Density\left(v_{1}^{2}\right)=\frac{5}{\;\left(5\times 4\right)\;/2}=0.2.$$

Table 1 summarizes the density of each node in two-hop subgraph of the sample network in Figure 2. As observed, the value of density is proportional to the CB certainty of the node, which is directly related to its location in the network. For instance, nodes 2, 6, node 12, which are marginal nodes in the network, exhibited high node density, while node 5, in the central part of the network, exhibited lowest node density. The real community structure has a similar characteristic: nodes with low node densities tend to occur with close connections to other communities, while nodes with high node densities exhibit no connections to other communities. This is the opposite to the node centrality in conventional community detections, and can be used for the determination of seed nodes for community division.

### 2.3. Similarity

In complex networks, the connections among intracommunity nodes are dense, while intercommunity nodes are sparse [24]. Node similarity is an effective parameter for the quantification of node affinity; the degree of similarity between two nodes is proportional to their common adjacent nodes, i.e., nodes with high similarity tend to connect to each other. So, the similarity of two nodes is a key parameter in the evaluation of the affinity of nodes *i* and *j* [25]. Node similarity includes common neighbors, Cosine, Jaccard, Sorensen index, PHI, Preferential attachment, Adamic-Adar, Allocation of resources [26,27,28,29,30,31,32,33], and Random walk similarities [34,35,36]. In this paper, we interpret similarity of $v_{i}$ and $v_{j}$ by calculating it based on their Jaccard correlation coefficients:

(4) $$JacSim\left(v_{i},\;v_{j}\right)=\frac{\left|N\left(v_{i}\right)\cap N\left(v_{j}\right)\right|}{\left|N\left(v_{i}\right)\cup N\left(v_{j}\right)\right|}\;$$

where $N\left(v_{i}\right)\;\mathrm{and}\;N\left(v_{j}\right)$ are adjacent node sets of node $v_{i}$ and *v_j_*, $N\left(v_{i}\right)\;\mathrm{and}\;N\left(v_{j}\right)$ $v_{i}$, $\left|N\left(v_{i}\right)\cap N\left(v_{j}\right)\right|$ refers to the quantity of common adjacent nodes shared by $v_{i}$. and *v_j_*, and $\left|N\left(v_{i}\right)\cup N\left(v_{j}\right)\right|$ refers to the quantity of nodes in the union of common adjacent node sets of $v_{i}$ and $v_{j}\;$.

### 2.4. Balance

It is well known that the selection of seed nodes with good centrality can improve the performance of *k*-means clustering. Centrality parameters including betweenness, closeness, k-shell, and uniform H-index have limitations in community detections [37]. The community centrality can precisely describe node centrality [38], and the computing complexity of community centrality is $O\;\left(nk^{5}\right)\;$. Despite this, the node degree centrality is a key parameter describing the community centrality in networks. Only the selection of seed nodes in *k*-means clustering algorithms based on node degree centrality may lead to overly-short distances between initial cluster centers, thus affecting clustering performance. As it can precisely reflect the CB certainty of nodes, the node density can be combined with the degree centrality as criteria for the selection of initial nodes. Therefore, $DD\left(v_{i}\right)\;$, the parameter for selection of the i -th initial node, is defined as:

(5) $$DD\left(v_{i}\right)=Density\left(v_{i}^{h}\right)\times Degree\left(v_{i}\right)$$

where h refers to the hop count of forward BFS, $Density\left(v_{i}^{h}\right)$ refers to the node density of $v_{i}$ calculated by Equation (3), and $Degree\left(v_{i}\right)$ refers to the node degree of $v_{i}\;$.

## 3. Method

In *k*-means clustering algorithms, the number of clusters is a key parameter. In [39], the Monte Carlo-based algorithm proposes an effective method by which to determine the community quantity. Hence, this study focuses on the effective selection of initial seed nodes and community detection in networks using *k*-means clustering algorithms in complex network with known community numbers.

As mentioned, node density is proportional to the CB certainty of a node in a specific community, and can be employed for the selection of seed nodes. However, the seed nodes cannot be selected based on the node density alone, as it may lead to the selection of isolated nodes, thus reducing the accuracy of clustering. Meanwhile, the seed nodes cannot be selected based on the degree centrality alone either, as most of the seed nodes selected in this way may be in same community due to the limited information contained in the degree centrality. Therefore, we propose DD , a parameter balancing node degree centrality and node density, as a criterion for initial node selection. 

In summary, the DDJKM algorithm based on node density, degree centrality, and conventional *k*-means clustering algorithms is proposed. In this algorithm, initial cluster centers are selected based on a combination of node degree, density, and similarity, while node centrality is also considered to avoid the selection of isolated nodes, thus avoiding local convergence in clustering and improving the effectiveness of community detection.

### 3.1. DDJKM Algorithm

Input: undirected connection network G={V,E}, the quantity of communities to be divided is K. V, and E are sets of nodes and edges.

Output: community division = Com (1), Com (2), …, Com (K).

Step 1: Establish the *n*-dimensional vector E (G) of the node degree and the *n*-dimensional vector  D(G) of node density based on $Density(v_{i}^{h})$:

(6) $$D(G)=(Density(v_{1}^{h}),Density(v_{2}^{h}),\ldots Density(v_{n}^{h}))$$

Step 2: All nodes in the network are arranged in descending order, $DD\left(v_{i}\right)\;$, which is the product of node density and node degree according to Equation (5). In cases of nodes with same $DD\left(v_{i}\right)\;$, these nodes are arranged in ascending order of node number. In this way, DDSeq(G), a sequence of $DD\left(v_{i}\right)$ of nodes in the entire network, is established;

Step 3: Select the first element in DDSeq(G) as the first initial node in the *k*-means clustering algorithm, add it to the clustering center node set Seed(v) , and obtain S(v) , which consists of nodes in the network that are not clustering center nodes:

(7) S(v)=G(v)−Seed(v)

where G(v) is the set of all nodes in network G.

Step 4: Calculate the node similarity using Equation (4) and establish the *n*-dimensional Jaccard (G) of nodes in network G:

(8) $$Jaccard\left(G\right)=\left[\begin{array}{ccc} JacSim\left(v_{1},\;v_{1}\right) & \cdots & JacSim\left(v_{1},\;v_{n}\right)\\ \vdots & \cdots & \vdots \\ JacSim\left(v_{n},\;v_{1}\right) & \cdots & JacSim\left(v_{n},\;v_{n}\right) \end{array}\right]$$

where $JacSim\left(v_{i},\;v_{j}\right)$ refers to the *Jaccard* correlation coefficient between $v_{i}$ and $v_{j}.$

Step 5: Calculate the correlation matrix DDJ(G) of nodes in network G using Equations (6) and (8):

(9) $$DDJ\left(G\right)=\left(D\left(G\right)D{\left(G\right)}^{T}\right)\;Jaccard\left(G\right)$$

where $D\left(G\right)D{\left(G\right)}^{T}$ is matrix product of D(G) and $D{\left(G\right)}^{T}\;$, and DDJ(G) is the Hadamard product of $D\left(G\right)D{\left(G\right)}^{T}$ and Jaccard(G) .

Step 6: Calculate the average correlation ($R_{p}\;$) of nodes in S(v) and nodes in Seed(v) :

(10) $$R_{p}=\sum_{q=1}^{\left|Seed\left(v\right)\right|}R_{qp}/\left|Seed\left(v\right)\right|$$

where $R_{qp}$ refers to the node correlation (correlation value in the correlation matrix DDJ(G) ) of $v_{p}$ and $s_{q}\;$, q=1, 2, …, |Seed(v)|, p=1, 2, …, |S(v)|, |Seed(v)| refers to the number of nodes in Seed(v) , and |S(v)| refers to the quantity of nodes in S(v) .

Step 7: Determine the minimum average correlation ($\mathrm{Min}R_{p}\;$) and establish MinMean(v) that consists of nodes in S(v) with average correlation = $\mathrm{Min}R_{p}\;$.

Step 8: Calculate $DD\left(v_{i}\right)\;$, which is the product of node density and node degree of each node in the node set MinMean(v), and add the node with the maximum $DD\left(v_{i}\right)$ to Seed(v) .

Step 9: If |Seed(v)| = *K*, terminate iteration; if not, return to Step 6.

Step 10: Execute the *k*-means community detection clustering algorithm.

Step 11: Export *K* communities (Com (1), Com (2), …, Com (K)) as each community corresponds to a clustering result.

### 3.2. K-Means Community Detection Clustering Algorithm

Input: K clustering centers, node similarity matrix Jaccard(G) .

Output: Cluster (1), Cluster (2), …, Cluster (K).

Step 1: The Euclidean distance of node similarity vector is:

(11) $$Jacd\left(jv_{a},\;jv_{b}\right)=\sqrt{\sum_{i=1}^{n}{\left(JacSim\left(v_{a},\;v_{i}\right)-JacSim\left(v_{b},\;v_{i}\right)\right)}^{2}}$$

where $jv_{a}$ and $jv_{b}$ refer to similarity vectors (in Jaccard (G) ) corresponding to $v_{a}$ and $v_{b}\;$. The Euclidean distance of other nodes to K clustering centers are inversely proportional to their similarity. Then, all nodes are categorized into the cluster whose clustering center has a shortest distance from this node. In this way, K clusters (Cluster (1), Cluster (2), …, Cluster (K)) are generated.

Step 2: Recalculate the clustering center of Cluster (*j*) and define it as a new clustering center $C_{j}\;$:

(12) $$C_{j}=\sum_{n=1}^{\left|Cluster\left(j\right)\right|}Jaccard{\left(G\right)}_{nj}/\left|Cluster\left(j\right)\right|$$

where $Jaccard\;\left(G\right){\;}_{nj}$ refers to the vector in Jaccard (G) corresponding to $v_{n}$ in the *j*-th cluster, 

n=1, 2, …, |Clustr (K)| , and |Cluster (j)| refers to the number of nodes in the *j*-th cluster.

Step 3: Calculate the Euclidean distances of all new and previous clustering centers to determine their maximum variation (MaxDist).

Step 4: If MaxDist remains unchanged or the maximum iteration times (Max-Iteration) were reached, iteration is terminated; proceed to the next step, otherwise return to Step 1.

### 3.3. Complexity Analysis

The complexity of community detection in this study is mainly caused by the density and community detections. In the calculation of density, the density of each node should be calculated. Meanwhile, we define the forward hop count as h , the average node density as $\bar{d}\;$, the total number of nodes in the network as n , and the time complexity in the process as $\;O\left(n{\bar{d}}^{h}\right)\;$. As the density calculation is a local process, it can be achieved by distributed computation; the time complexity is $O\left({\bar{d}}^{h}\right)$ where h≤3 in most cases. The DDJKM algorithm involves the calculation of a correlation degree matrix DDJ(G), which is a sparse matrix. Meanwhile, $D\;\left(G\right)\;\times D\;{\left(G\right)}^{T}$ is a sparse matrix whose calculated complexity does not exceed O(m) . In community detection, the degree and local similarity of each node should be obtained, taking O(mn) operations to traverse all edges and adjacent nodes, where m is the number of edges. The complexity of the *k*-means algorithm is O(nKt), where K refers to the cluster quantity and t to the iteration times. As K≪n and t≪n in most cases, the complexity of DDJKM algorithm is $O\left({\bar{d}}^{h}+mn+nKt+m\right)=O\left(mn\right).\;$

## 4. Experimental

In this section, we used seven real network datasets and the LFR benchmark datasets to validate the performance of the proposed algorithm. The real-world networks include Zachary’s karate club network [40], the Dolphin social network [41], Books about US politics network [42,43], the American college football network [44], the Amazon copurchase network [45], and the YouTube network [45]. LFR benchmark networks possess properties found in real-world networks, such as heterogeneous distributions of degree and community size. First, we present some commonly-used evaluation measures. Then, we explain the real network and computer-generated networks we use, and compare our algorithm with some known algorithms.

### 4.1. Evaluation Measures

Normalized mutual information (NMI) is taken as the performance measure. NMI reflects the similarity between the true community and the detected community structures. Given two parts, A and B, of a network, C is the confusion matrix. In C , $C_{ij}$ is the number of nodes of community i of part A that are also in community j of part B [46]. NMI I(A,B) is defined as follows [47]:

(13) $$I\left(A,B\right)=\frac{-2\sum_{i=1}^{C_{A}}\sum_{j=1}^{C_{B}}C_{ij}log\left(\frac{C_{ij}\cdot N}{C_{i}\cdot C._{j}}\right)}{\sum_{i=1}^{C_{A}}C_{i}\cdot log\left(\frac{C_{i}}{N}\right)+\sum_{j=1}^{C_{B}}C._{j}\cdot log\left(\frac{C._{j}}{N}\right)}$$

where, $C_{A}\left(C_{B}\right)$ is the number of classes in part A(B) , $C_{i}\cdot \left(C._{j}\right)$ is the number of elements of C in row i (column j ), and N is the total number of nodes. If A=B , I(A,B)=1 ; if A and B are totally different, I(A,B)=0 . As NMI increases, the detected communities become more approximate to the true communities.

Given a network G=(V,E) , let T be the set of ground-truth communities and D be the set of communities detected by the community detection algorithm. Each ground-truth community $T_{i}\in T\;\;$(or each detected community $D_{i}\in D\;$) is a set consisting of the member nodes. Average F1 score is a popular metric to evaluate the degree of similarity between two sets. When applied in community detection, it can be formed as [48].

(14) $$F1\left(T,D\right)=\frac{1}{2}\left(\frac{1}{\left|T\right|}\sum_{T_{i}\in T}F\left(T_{i},D\right)+\frac{1}{\left|D\right|}\sum_{D_{j}\in D}F\left(D_{j},T\right)\right)$$

where

(15) $$F\left(T_{i},D\right)=\underset{D_{j}\in D}{\max}F1\left(T_{i},D_{j}\right)$$

and $F1\left(T_{i},D_{j}\right)$ is the harmonic mean of precision and recall. The formulation of $F\left(D_{j},T\right)$ can be expressed in the same way.

### 4.2. Testing Networks

#### 4.2.1. Real-World Networks

**Figure 3:**
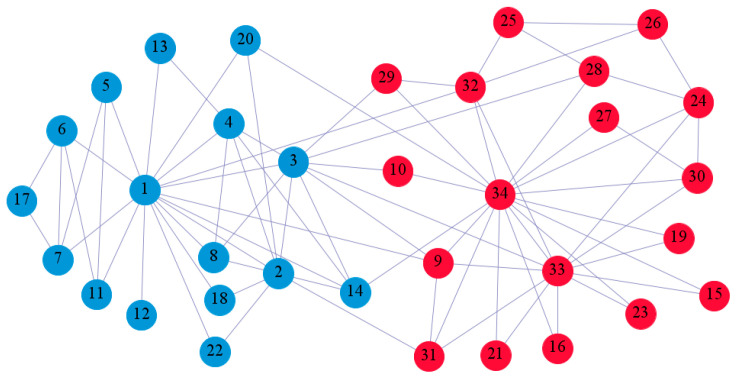
Zachary’s karate club.

**Figure 4:**
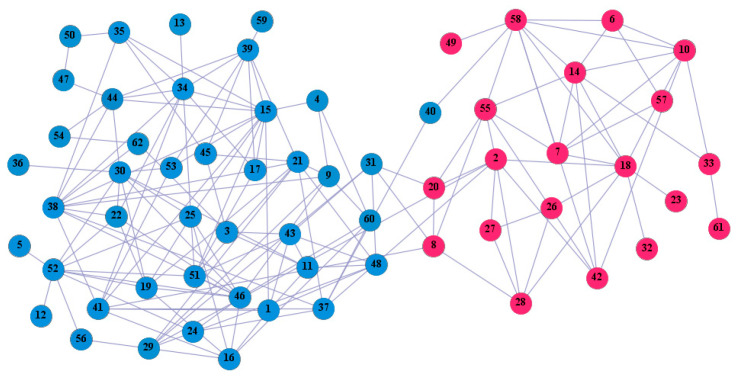
Dolphin social network.

**Figure 5:**
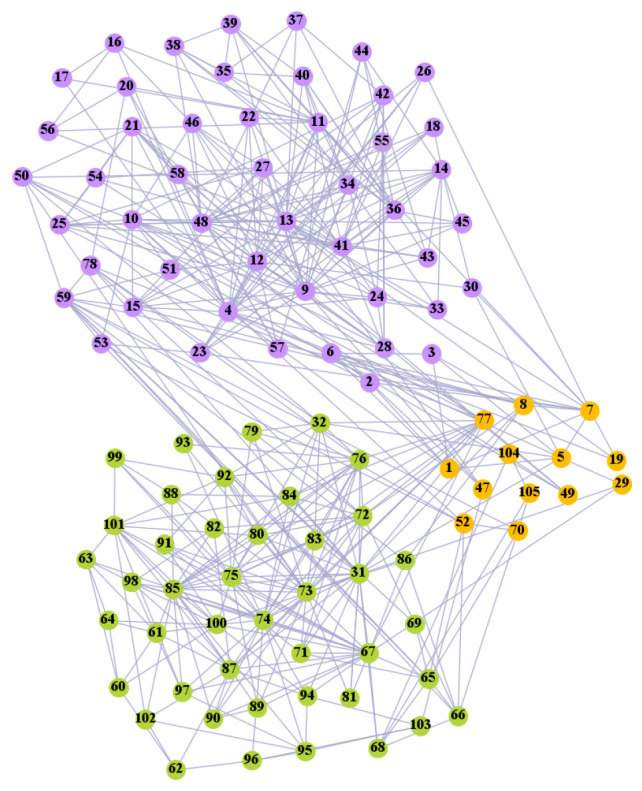
Books about US politics.

**Figure 6:**
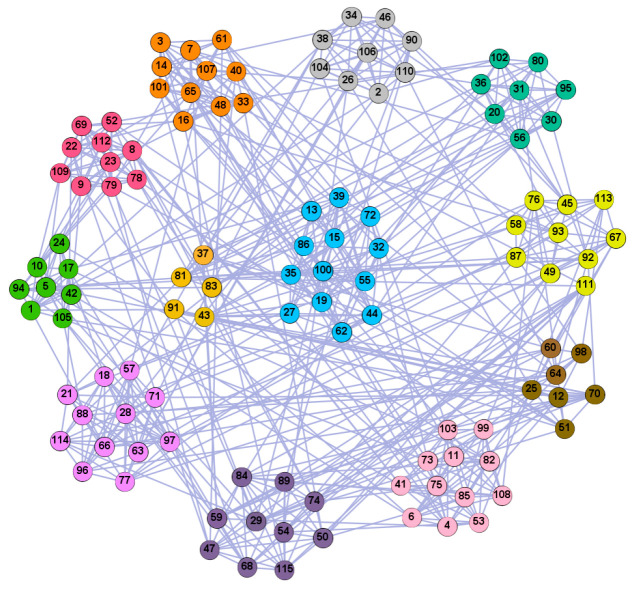
American College football.

In the following part, we provide a simple description of the real network used in the experiments. For all these networks, the community structure is recognized which makes them suitable to evaluate the community detection methods. Zachary’s karate club [40] is one of most the widely-used networks in community detection. The 34 members of the club constitute the 34 nodes of the network. The relationships between members constitute the 78 edges of the network. The Dolphin social network [41], proposed by Lusseau, is shown in Figure 3. The connection of any two dolphins represents a tighter connection between them. The dolphin social network consists of 62 dolphins as the nodes and 159 connections as the edges. The network can be detected as two communities, as shown in Figure 4. The Books about US politics [42,43] network consists of 105 books about US politics published in 2004 and sold by amazon.com. Based on the descriptions and reviews of the books posted on Amazon, Newman divided the network into three communities. The network is shown in Figure 5. The American college football [44] network was proposed by Girvan and Newman. The nodes represent different football teams, and the edges represent the matches between them. The network consists of 115 nodes and 616 edges. The network consists of 12 communities comprising 12 football teams. The network is shown in Figure 6. The Amazon copurchase and YouTube networks are provided by SNAP [45].

#### 4.2.2. Computer-Generated Network

We tested our algorithm on LFR benchmark networks which were proposed by Lancichinetti et al. [49]. The LFR generation program provides a rich set of parameters through which the network topology can be controlled, including network size N , the average degree 〈k〉, the maximum degree $k_{max}\;$, the minimum and maximum community size, $C_{min}$ and $C_{max}\;$ respectively, and the mixing parameters μ . The node degrees are governed by power laws with exponents of $\tau_{1}$ and $\tau_{2}\;$. In this work, we employ four types of LFR networks with scales of 1000 (LFR1), 2000 (LFR2), and 5000 (LFR3, LFR4) nodes with other corresponding parameters, as shown in Table 2.

### 4.3. Experimental Results and Analysis

In this study, the performance of the proposed algorithm was evaluated using five real-world networks and LFR networks. According to the small world effect, which indicates that the average minimum route between any two nodes in a complex network is 6, *h* in the forward BFS shall be set as 3 to achieve optimized performance. The criteria for iteration termination in the proposed algorithm are consistent with those in conventional *k*-means algorithms, i.e., once the Euclidean distances of new and previous clustering center vectors remain unchanged, iteration is terminated, indicating convergence at constant clustering, which is defined as one of the iteration termination conditions. Meanwhile, the Max-Iteration variable was set to 100 since the maximum number of observed in this paper iterations was 20. Therefore, the network parameters in this study were determined based on *h* = 3 and Max-Iteration = 100.

#### 4.3.1. Experiments on Real-World Networks

**Figure 7:**
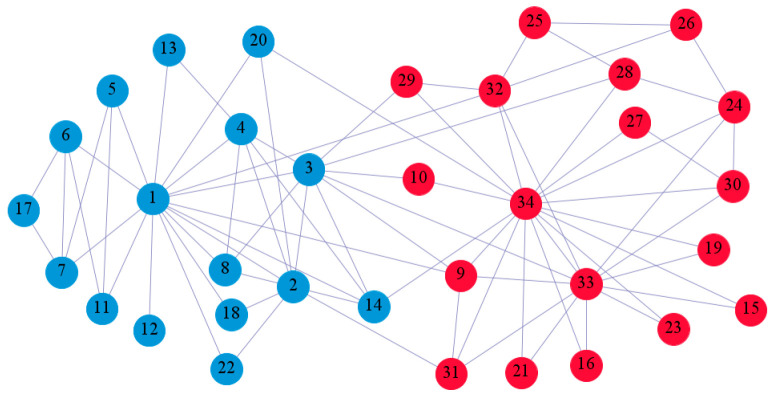
The community structure of the Zachary’s karate club network as detected by the proposed method.

**Figure 8:**
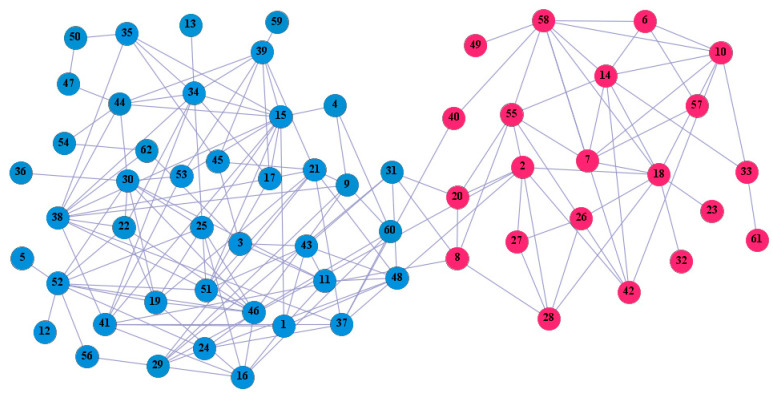
The community structure of the Dolphin social network as detected by the proposed method.

**Figure 9:**
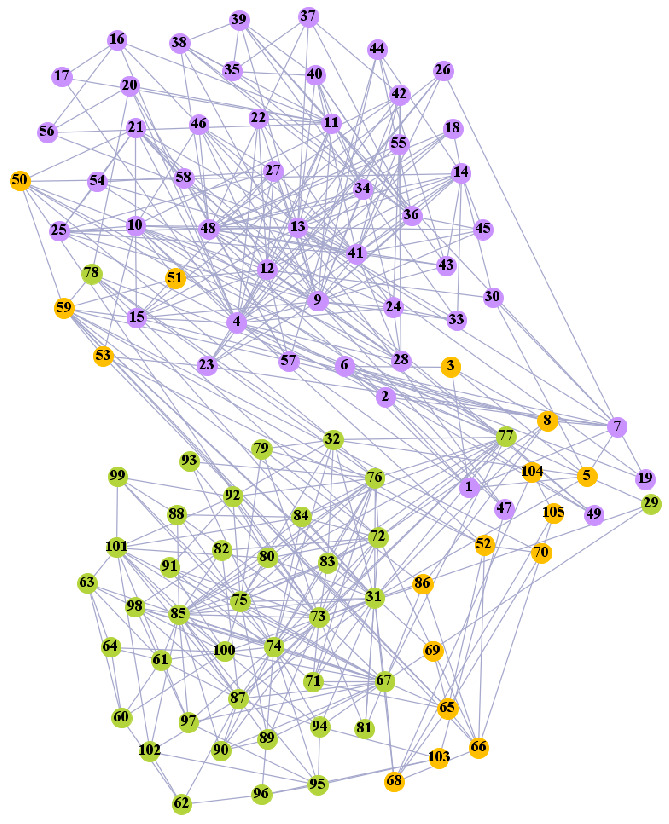
The community structure of the Books about US politics network as detected by the proposed method.

**Figure 10:**
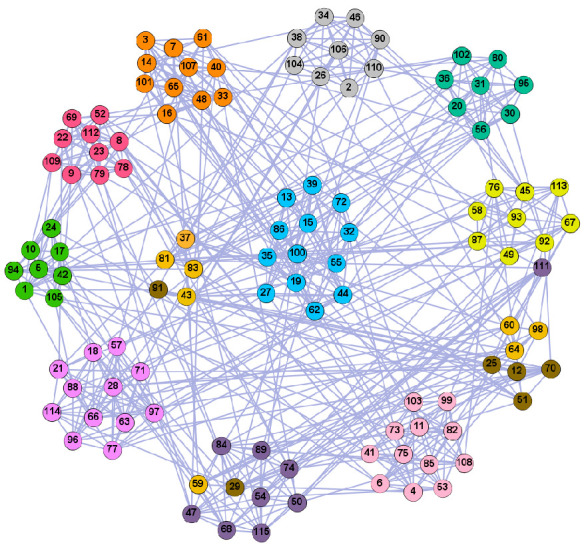
The community structure of the American college football network as detected by the proposed method (12 communities).

**Figure 11:**
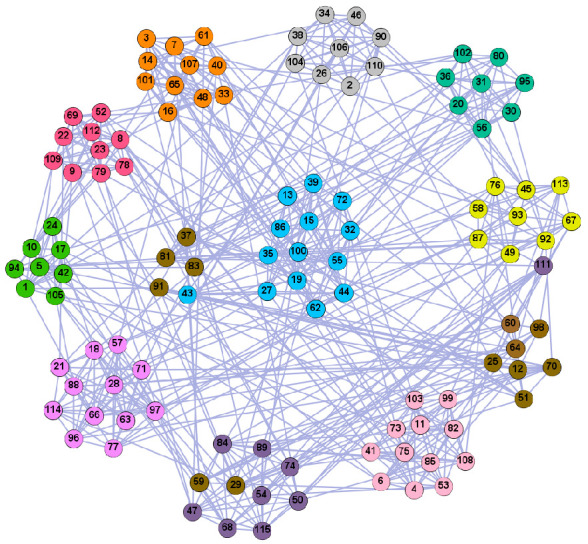
The community structure of the American college football network as detected by the proposed method (11 communities).

We used the five real-world networks mentioned above to verify the efficiency of our algorithm. As shown in Figure 3; Figure 7, the final community structure of the Zachary’s karate club network detected by DDJKM was consistent with the actual structure. It can be seen from Figure 4 and Figure 8 that the structure in the Dolphin social network detected by our algorithm is also very close to the actual structure. Only node 40 is misidentified by our algorithm, and it can be seen that node 40 is in close proximity to two communities. The results for the Books about US politics network detected by our algorithm are shown in Figure 9. In the American college football network, our algorithm divides it by 12 (Figure 10) and 11 (Figure 11). Compared with the results shown in Figure 6, we can see that our algorithm performs well on the American football network; most nodes are correctly classified into their actual community structures.

We compared the performance of our algorithm with the GN algorithm [24], the Newman fast greedy algorithm (FG) [50], the sparse linear coding method (SLC) [51], the MIGA algorithm [52], the Equation (20) algorithm [53], and the k-means algorithm in Section 3.2 on real-world networks. The results are presented in Table 3. The F1-score (F1) and Normalized mutual information (NMI) were used to compare our algorithm with the reference algorithms. Our algorithm performed well on most of the networks. Furthermore, the algorithm grouped most of the nodes into the correct communities and the normalized mutual information value (NMI) reached 0.933 and 0.923, respectively, when 11 and 12 communities were divided in the American college football network.

We use the top-5000 ground-truth communities of the Amazon copurchase and the YouTube networks provided by SNAP [45]. We compared the experimental results of our proposed algorithm with the weighted version of LPA (WLPA) [48] on these real-world networks. As shown in Table 4, we can see that the DDJKM algorithm performed well. The score of DDJKM on the Amazon network is slightly lower than of WLAP, but its score on the YouTube is higher than that of WLAP, and the mixing (μ) of the YouTube network is higher than the Amazon network, i.e., up to 0.840, which indicates that our algorithm can also achieve good community detection results on a highly-mixed network.

#### 4.3.2. Experiments on LFR Benchmark Networks

Next, we used LFR networks LFR1, LFR2, and LFR3 to test DDJKM and the *k*-means algorithm described in Section 3.2. Because the results of the *k*-means algorithm are different each time, we took the average of the results of the above three networks and ran them 20 times using these algorithms.

**Figure 12:**
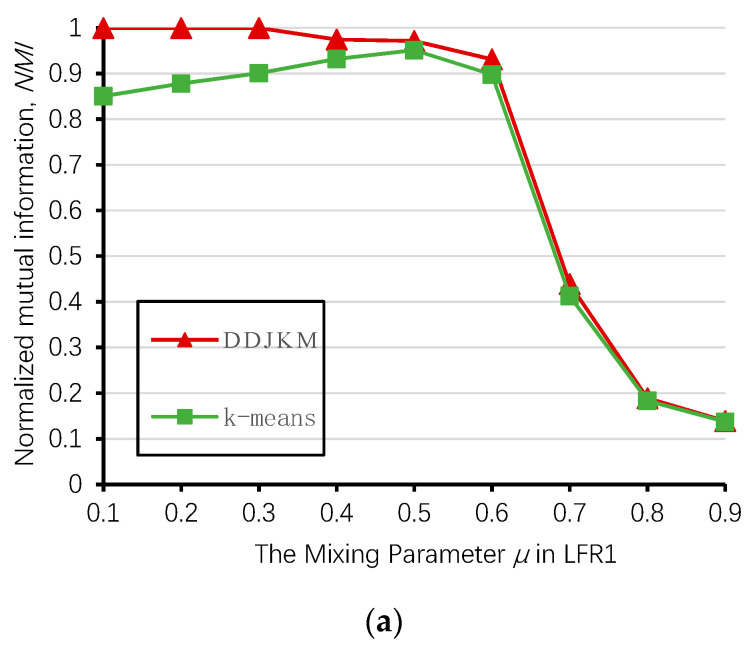

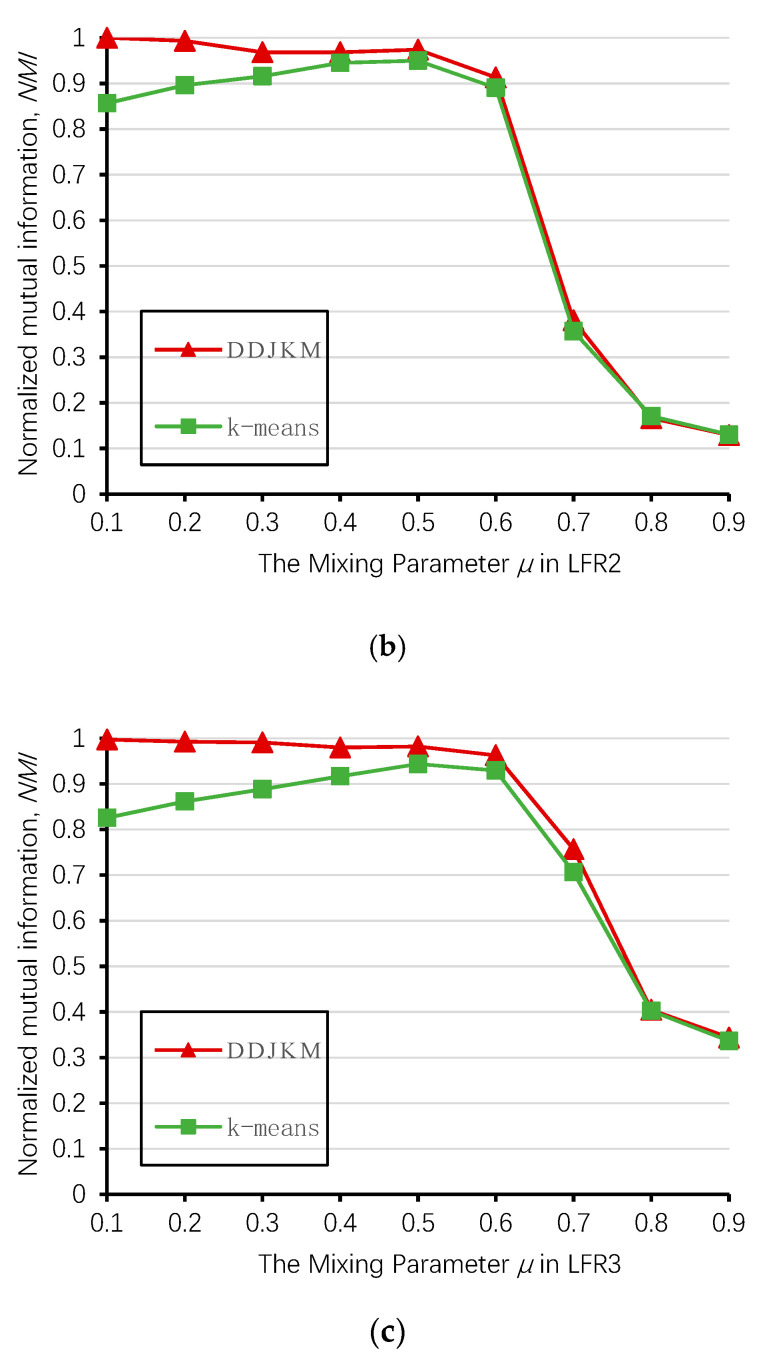
Values of NMI over the 20 runs on (**a**) LFR1, (**b**) LFR2, and (**c**) LFR3.

Figure 12 shows the results of our algorithm and the *k*-means algorithm on the LFR1, LFR2, and LFR3 networks; the DDJKM results showed the best performance. The DDJKM algorithm performs well in the range of μ < 0.6, and with an increase of μ , the DDJKM algorithm was stable on the LFR network of 1000, 2000, and 5000 nodes, and there is no significant difference in the performance of the network with different numbers of nodes and community scales. This means that the DDJKM algorithm is stable in the dense network, and is not affected by the number of nodes or the community scale. However, when μ > 0.6, the NMI value of DDJKM and the *k*-means algorithms running on the three computer-generated networks suddenly drop everything, because the community structure is less obvious as the mixing parameters increase, causing too many nodes to merge into the same community. Therefore, the accuracy of the algorithms continues to decrease.

**Figure 13:**
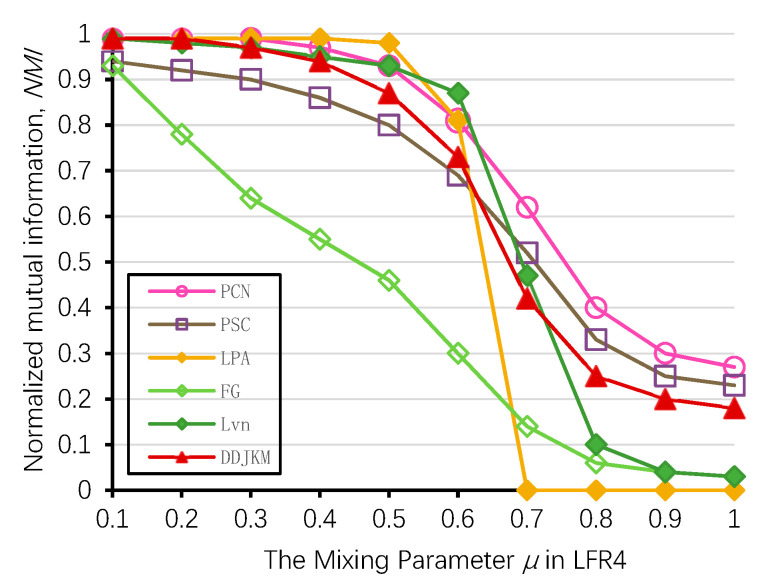
Comparison of our method and known algorithms on LFR4.

On the LFR (LFR4) network of 5000 nodes, we ran some of the known community detection algorithms, i.e., Newman’s fast greedy algorithm (FG), Louvain (Lvn) [10], Label Propagation (LPA) [12], PCN, and PSC [54] and compared their results with the results of our algorithms. We generated 100 LFR networks per μ value, ran the algorithms on all the 100 generated datasets, and averaged the results for each algorithm. The results of the NMI performance are shown in Figure 13. We present the detailed results of the algorithms on the LFR4 networks of 5000 nodes in Table 5. On the networks generated with higher mixing values (i.e., μ. > 0.8), our algorithm with PCN and PSC was among the top four best performing algorithms according to the NMI values; our algorithm has slightly lower accuracy than PCN and PSC when the mixing parameters are high; on most networks, PCN, PSC, and our algorithm yield the best results; Newman’s algorithm and the Louvain algorithm only have higher NMI values when the mixing value is low, as they tend to merge communities which may lead to a resolution limit [55]. The NMI value of LPA is relatively high when the mixing value is low in a large-scale network. However, with the increase of mixing values, the community structure is less obvious, and its accuracy is significantly reduced. Our algorithm can still successfully identify the community, and its performance is better than Newman’s greedy fast algorithm, Louvain, and LPA.

## 5. Conclusions

In this study, the concepts of CB uncertainty of nodes based on information entropy and of CB certainty of nodes as node density were defined. In addition, based on node density and degree centrality, a k-means clustering-based community detection algorithm, DDJKM, was proposed. This algorithm can select clustering centers well, thus preventing the selection of initial cluster centers which are too close to each other, and reducing the iteration times in the process. The proposed algorithm exhibited good performance in several representative, real-world networks, as well as in artificial networks. In future works, as the node density can reflect its community belongingness, nodes can be divided into two categories, i.e., with CB certainty and with CB uncertainty, so that study of community detection can focus on the detection of nodes with CB uncertainty. In this way, the number of required iterations for the community division of nodes can be effectively reduced.

## Figures and Tables

**Table 1:** CB uncertainty of each node in the two-hop subgraph on the sample network shown in Figure 2.

Node	Density
2, 6, 12	0.667
1, 3, 7, 9, 11, 13	0.5
4, 8, 10	0.333
5	0.2

**Table 2:** Parameter settings of LFR benchmark networks.

Network	N	$\;\tau_{1}\;$	$\;\tau_{2}\;$	$\;C_{min}\;$	$\;C_{max}\;$	〈k〉	$\;k_{max}\;$	μ
LFR1	1000	2	1	20	50	20	50	0.1-0.9
LFR2	2000	2	1	20	100	20	50	0.1-0.9
LFR3	5000	2	1	20	50	20	50	0.1-0.9
LFR4	5000	2	1	20	100	15	75	0.1-1.0

**Table 3:** Experimental results ( F , NMI) of the community detection algorithm. The best results are marked in bold.

Network		GN	FG	MIGA	SLC	Equation (20)	*k*-means	DDJKM	
Karate	|c|	2	2	2	2	2	2	2	
	F1	0.970	0.971	**1**	0.971	**1**	0.879	**1**	
	NMI	0.836	0.837	**1**	0.837	**1**	0.666	**1**	
Dolphins	|c|	2	2	2	2	2	2	2	
	F1	0.980	0.937	0.965	0.980	0.961	0.770	**0.982**	
	NMI	**0.890**	0.652	0.814	**0.890**	0.814	0.417	0.889	
Polbooks	|c|	3	3	3	3	3	3	2	3
	F1	0.808	0.725	0.797	0.798	**0.829**	0.655	0.784	0.726
	NMI	0.568	0.568	0.585	**0.584**	0.597	0.454	0.571	0.530
Football	|c|	12	12	12	12	12	12	11	12
	F1	0.802	0.528	0.864	0.846	0.859	0.730	**0.920**	0.885
	NMI	0.878	0.697	0.916	0.793	0.865	0.822	**0.933**	0.923

**Table 4:** Experimental results (F, NMI) of the community detection algorithm. The best results are marked in bold.

Network		WLPA	DDJKM
Amazon	F1	**0.582**	0.554
	NMI	**0.761**	0.755
YouTube	F1	0.273	**0.482**
	NMI	0.547	**0.625**

**Table 5:** Generated LFR benchmark networks of 5000 (LFR4) nodes.

|V|	μ	NMI					
		DDJKM	PCN	PSC	LPA	FG	Lvn
5000	0.1	0.99	0.99	0.94	0.99	0.93	0.99
5000	0.2	0.99	0.99	0.92	0.99	0.78	0.98
5000	0.3	0.97	0.99	0.90	0.99	0.64	0.97
5000	0.4	0.94	0.97	0.86	0.99	0.55	0.95
5000	0.5	0.87	0.93	0.80	0.98	0.46	0.93
5000	0.6	0.73	0.81	0.69	0.81	0.30	0.87
5000	0.7	0.42	0.62	0.52	0.00	0.14	0.47
5000	0.8	0.25	0.40	0.33	0.00	0.06	0.10
5000	0.9	0.20	0.30	0.25	0.00	0.04	0.04
5000	1.0	0.18	0.27	0.23	0.00	0.03	0.03
